# Supplementary material for: The Contribution of Registered Dietitians in the Management of Hyperemesis Gravidarum in the United Kingdom
Source: Nutrients. 2021 Jun 8;13(6):1964. doi: 10.3390/nu13061964 (PMC8226879; doi:10.3390/nu13061964)
Supplement: Supplementary file 1 [file nutrients-13-01964-s001.zip › nutrients-1244236-supplementary.pdf]

Supplementary File S1: Survey

Thank you for agreeing to complete this survey. It will take approximately 10-12 minutes to complete.

Background: Hyperemesis Gravidarum (HG) is a condition at the severe end of the pregnancy sickness spectrum. Pregnancy nausea and vomiting exist on a continuum, with no specific point at which it becomes HG, making diagnosis, clinical management and research challenging.

The definition of HG varies, but typically involves persistent and intractable nausea and vomiting with the triad of more than 5% pre-pregnancy weight loss, dehydration and electrolyte imbalance.

In the context of this questionnaire, we are keen to understand more about the role dietitians play in the management of women **with these severe symptoms**, even if they have not been labelled with a formal diagnosis of HG.

Screening questions:

A. **Are you a registered dietitian currently practising in the United Kingdom?** Yes/No
B. **Have you ever had a woman referred to you for dietetic advice due to Hyperemesis Gravidarum (severe or persistent nausea and vomiting in pregnancy)?** Yes/No
C. **Do you consent to take part in this study? (***Participation is voluntary and you may leave the questionnaire at any time without saving your responses, however once you complete the questionnaire it will not be possible to withdraw your data as the information collected is anonymous*) Yes/No

Main questionnaire

1. Does your department have a service level agreement to provide dietetic cover to maternity care/obstetrics patients?
   - Yes
   - No
   - Don't know

   a. If yes, how much time (full time equivalent) is allocated per week? _____________hours/week

2. What setting do you work in? *(please select one option)*
   - Secondary care NHS hospital
   - Tertiary care NHS hospital
   - Specialist NHS maternity hospital
   - Private hospital
   - Other, please state ______________

3. Which part of the UK do you work in? *(please select one option)*
   - England
   - Scotland
   - Wales
   - Northern Ireland

4. Approximately how long have you been working as a dietitian? *(please select one option)*
   - <2 years

- 2-5 years
- 6-10 years
- >10 years

5. What is your main dietetic role/specialism? *(please select one option)*
    - General dietitian/ newly qualified dietitian
    - Gastroenterology
    - Nutrition support team
    - Maternal health dietitian
    - Other, please state________________

6. In what setting do you usually see women referred for HG? *(please tick all that apply)*
    - Inpatient
    - Outpatient
    - Day case setting (e.g. maternity day unit)
    - Other, please setting___________________

7. Please estimate approximately how many women are referred to you per year regarding HG?
   ____________________ per year

8. Does your employment setting have a day unit rehydration service for women with HG?
    - Yes
    - No, but there is currently a service being developed
    - No
    - Not sure

9. Which department/health professional usually makes the referral to dietetics? (*please tick all that apply*)
    - Obstetrician
    - Other medical doctor
    - Midwife
    - Nurse
    - General practitioner
    - Other, please state ______________________

10. What criteria is most commonly used for referring a women with HG to your dietetic service? (*please tick all that apply*)
    - Malnutrition Universal Screening Tool (MUST)
    - Pregnancy Unique Quantification of Emesis score (PUQE)
    - Percentage weight loss, please state __________% used
    - Referral is made after more than one admission for pregnancy sickness
    - Ketone level
    - No specific criteria used
    - Other, please state_______________________

11. Do you follow a defined clinical pathway/guideline for dietetic management of women with HG? *(please select one option)*
    - Yes, a local pathway/guideline – please see q24 if you would be willing to share your guideline with us.
    - Yes, a national guideline – please state name ________________________________
    - No specific guideline used

12. How frequently do you use oral nutritional supplements (ONS) in women with HG? *(please select one option)*
    - Never
    - Rarely
    - Sometimes
    - Most of the time
    - All of the time

    12a. If used, what type of ONS do you most commonly recommend?

    - Standard ready to feed milk-based supplement
    - Concentrated ready to feed milk-based supplement
    - Powdered milk-based supplement
    - Juice based supplement
    - Other, please state__________________

13. How frequently is enteral feeding (i.e. nasogastric tube feeding) used in women with HG under your care? *(please select one option)*
    - Never
    - Rarely
    - Sometimes
    - Most of the time
    - All of the time

    13a. If used, what type of enteral feeding is most common?

    - Nasogastric tube feeding
    - Nasojejunal tube feeding
    - Other type, please state ___________________

14. How frequently is total parenteral nutrition (i.e. feeding via a central vein rather than a peripheral vein) used in women with HG under your care?
    (please select one option)
    - Never
    - Rarely
    - Sometimes
    - Most of the time
    - All of the time

    14a. If total parenteral nutrition is not used, what are the reasons?(*please tick all that apply*)

- Concerns about central line insertion
- Concerns about risks of line infection and how that would be managed if it occurred
- Lack of training in maternity unit (i.e. midwives not trained to administer TPN)
- Unlikely to need TPN for a 'sufficient time' (e.g. > 5-7 days) for risks to outweigh the benefits
- TPN services not funded in maternity units
- Other reason, please state____________________

15. Do you consider refeeding syndrome guidelines in the dietetic management of HG? *(please select one option)*
    - Yes, all of the time
    - Yes, some of the time
    - No, refeeding syndrome is not usually relevant

16. Do you provide any patient resources to women with HG? *(please select one option)*
    - Yes, generic nutrition support resources
    - Yes, specific resources for pregnancy sickness/HG
    - No specific resources are used

17. Do you routinely offer a follow up service for women with HG once discharged from hospital? *(please select one option)*
    - Yes, always
    - Yes, sometimes
    - No

    a. If so, what follow up service do you usually offer?
       - Telephone review
       - Face to face appointment (prior to covid 19)
       - Other, please state____________________

18. Do you signpost women referred to you for HG to other services/other resources?
    - Yes, please state details __________________
    - No

19. What, if any, resources would you find useful in supporting women with HG? *(please tick all that apply)*
    - Specific patient resources
    - A mobile phone application for HG
    - Training for dietitians
    - Training for other healthcare professionals
    - A patient support group
    - Webinars
    - Other, please state__________________________________

20. Please rate how competent you *consider yourself to be* in the dietetic management of HG on a scale from 1 to 10, where 1 = not at all competent and 10 = very competent.
    1_______2________3_______4_______5________6______7_______8_______9_______10

Not at all competent Very competent

21. Do you have any suggestions or comments about what would optimise the referral rate/dietetic management of women with HG? (free text box) *(optional)*

22. In the future we may wish to contact dietitians to discuss their involvement in the development of HG guidelines or their practice in general. If you would like to potentially be contacted about future studies, please enter your details here (name, place of work, email address). *(optional)*

23. If you are happy to share any clinical guidelines or resources with us, please attach documents below. If you would prefer for these to be anonymous, please remove details of your organisation. *(optional)*

24. Please tell us how you heard about this study? *(please select one option)*

- Via the BDA Maternal and Fertility Nutrition Group
- Via the BDA Gastroenterology Specialist group
- Via the BDA PENG group
- From my manager
- From a colleague
- Via social media
- Other source, please state___________

Thank you for completing the questionnaire. If you have any questions about this study, please contact Dr Kate Maslin (kate.maslin@plymouth.ac.uk).
